# Metabolism of Histone Deacetylase Proteins Opsonizes Tumor Cells to Checkpoint Inhibitory Immunotherapies

**DOI:** 10.20900/immunometab20200002

**Published:** 2019-12-04

**Authors:** Paul Dent, Laurence Booth, Andrew Poklepovic

**Affiliations:** 1 Department of Biochemistry and Molecular Biology, Virginia Commonwealth University, Richmond, VA 23298-0035, USA; 2 Department of Medicine, Virginia Commonwealth University, Richmond, VA 23298-0035, USA

**Keywords:** autophagy, apoptosis, chaperone, drug, endoplasmic reticulum, ERK, histone deacetylase, immunotherapy, kinase, LC3-associated phagocytosis, MAP kinase, neratinib, off-target effect, pazopanib, pemetrexed, receptor tyrosine kinase, sildenafil, survival signaling

## Abstract

LC3-associated phagocytosis, a distinct form of autophagy, plays a key role in antigen presentation. Autophagy itself plays a central role in the regulation of cellular metabolism. Proteins that regulate autophagy include the AMPK which senses high levels of AMP, and mTOR, which integrates amino acid and fatty acid metabolism with autophagy. More recently, autophagy has been demonstrated to regulate tumor cell immunogenicity via the degradation of histone deacetylase proteins. Individual drugs and drug combinations that activate the ATM-AMPK pathway and inactivate mTOR, cause autophagosome formation. The maturation of autophagosomes into autolysosomes causes the autophagic degradation of histone deacetylase proteins who regulate the transcription of PD-L1, Class I MHCA, ODC and IDO1. Indeed, drug combinations that do not contain an HDAC inhibitor can nevertheless act as de facto HDAC inhibitors, via autophagic degradation of HDAC proteins. Such drug combinations simultaneously kill tumor cells via immunogenic autophagy and in parallel opsonize tumor cells to checkpoint inhibitor immunotherapies via reduced expression of PD-L1, ODC and IDO1, and increased expression of Class I MHCA.

## INTRODUCTION

**Figure 1.**
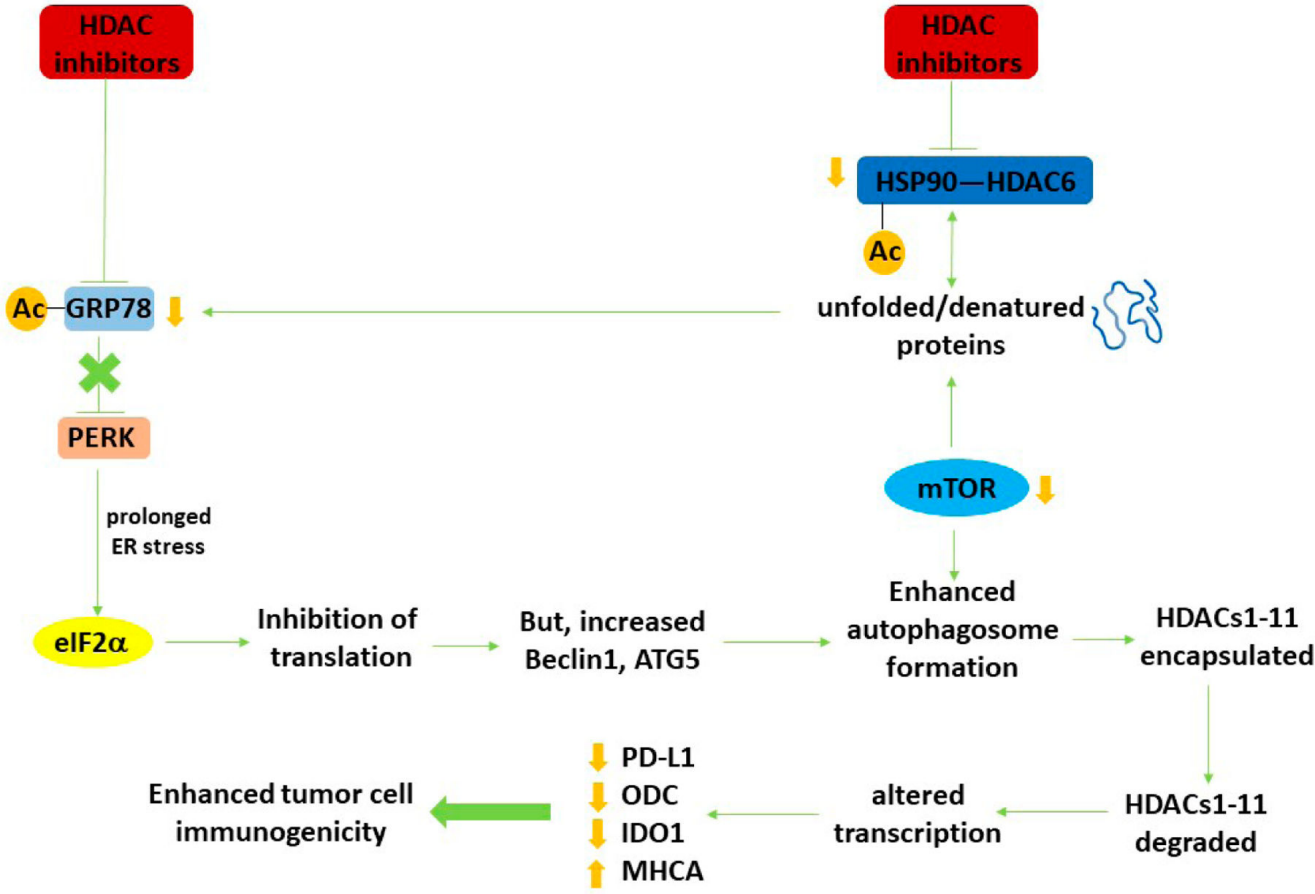
Autophagy regulates the expression of HDAC proteins which in turn leads to altered transcription and enhanced tumor cell immunogenicity. HDAC inhibitors increase the acetylation of multiple protein chaperones, thereby reducing chaperone function. Reduced chaperone function increases the levels of denatured proteins, including growth factor receptors and signaling intermediates, leading to inactivation of the autophagy master regulator mTOR. In parallel, because of the abundance of denatured proteins and the reduced functionality of GRP78, an endoplasmic reticulum stress response from PERK to eIF2α to reduced protein translation occurs. Reduced GRP78 function also results in activation of the IRE1 ER stress pathway which via activation of the c-Jun NH2 terminal kinase pathway can also facilitate tumor cell death. Although translation from the majority of genes is reduced, some, such as the autophagy regulatory proteins Beclin1 and ATG5 is enhanced. With the inactivation of mTOR and increased levels of Beclin1 and ATG5, autophagosome formation is enhanced. Encapsulated HDAC proteins in the autophagosome, following fusion with acidic endosomes to become autolysosomes, are degraded. Reduced expression of HDACs alters the transcription of many genes, notably that the protein levels of PD-L1, ODC and IDO-1 decline and that of Class I MHCA is enhanced. *In vivo*, these changes result in enhanced efficacy of checkpoint inhibitory immunotherapeutic antibodies. Adapted with permission from [16], copyright © 2019 Elsevier B.V.

It is now well-recognized that LC3-associated phagocytosis (LAP), a specialized form of autophagy, causes the ingestion of non-self extracellular proteins whose breakdown product peptides are then used for antigen presentation on the cell surface [1–5]. The process is also essential for the efficient clearance of dead cells. However, this form of immune cell protein metabolism is not the only mechanism by which the anti-tumor effects of the immune system can be stimulated via autophagy. This review will discuss how autophagy, a cellular process evolved to maintain cellular homeostasis, also can indirectly regulate tumor cell immunogenicity (Figure 1).

Several years ago, we demonstrated that GI tumor cells were killed by the multi-kinase inhibitor sorafenib combined with the HDAC inhibitors vorinostat or sodium valproate, in part, via CD95 death receptor signalling and autophagosome formation; this data was translated into two clinical trials (; ). Similar data in sarcoma cells using the multi-kinase inhibitor pazopanib combined with the novel HDAC inhibitor AR42 also resulted in a clinical trial (). Others have also demonstrated that autophagy is frequently a mechanism by which GI tumor cells can be killed by anti-cancer drugs, using the chemically dissimilar HDAC inhibitor panobinostat [6–8].

## HISTONE DEACETYLASE INHIBITORS AND HISTONE DEACETYLASE PROTEINS

For two decades it has been understood that histone deacetylase proteins (HDACs) regulate chromatin condensation and the transcription of genes [9–12]. Simplistically, by deacetylating histones associated with DNA, the inhibitors of HDACs should facilitate greater condensation and increased transcription. However, chemical inhibitors of HDACs were instead found to cause activation of some genes and for others to be inhibited. With the advent of checkpoint inhibitory immunotherapeutic antibodies, the possibilities of utilizing HDAC inhibitors to alter tumor cell immunogenicity via altered protein expression were clearly apparent [13–15]. For example, could HDAC inhibitors reduce the levels of PD-L1, ornithine decarboxylase and indoleamine 2,3-dioxygenase 1, and increase the expression of Class I MHCA? And, subsequently multiple clinical trials over the past 10 years have been and are listed combining HDAC inhibitors with immunotherapeutic antibodies (, , , , , , , , , , , , , , , , , ).

## THINKING OUTSIDE OF THE BOX FOR TARGETED DEGRADATION VIA AUTOPHAGY

Several years ago, using the FDA-approved irreversible ERBB1/2/4 inhibitor neratinib, we discovered by examining our control total protein loading data for ERBB1, ERBB2 and ERBB4 that not only did neratinib reduce receptor tyrosine phosphorylation, but it also reduced receptor protein levels [17]. This degradation effect was enhanced when neratinib was combined with HDAC inhibitors and was prevented by molecular knock down of ATG5 or Beclin1, *i.e*., the receptors were being degraded via autophagy. What was more surprising were data showing that our negative control receptors, c-MET and c-KIT, also were being degraded. Nota bene: degradation of ERBB1 required ubiquitination and autophagy whereas degradation of c-MET was independent of ubiquitination. We went on to determine that the combination of neratinib and HDAC inhibitors reduced the expression of plasma membrane localized mutant RAS proteins, and that this process of receptor/RAS internalization and degradation was dependent upon the protein Rubicon, *i.e*., LC3-associated phagocytosis was required for plasma membrane protein degradation effects [16,18–21]. These findings also have implications for the immune cells themselves. Neratinib, via inhibition of the MAP4Ks MST2/MST3/MST4, was shown to down-regulate RAS proteins not only in carcinoma cells but also in blood cancer cells [21].

In parallel to this project, another series of studies was defining the molecular mechanisms by which the thymidylate synthase and lung cancer therapeutic pemetrexed interacted with the phosphodiesterase 5 inhibitor sildenafil (Viagra) to kill non-small cell lung cancer cells. Because of the efficacy of checkpoint inhibitory immunotherapy antibodies in this disease, we also determined whether our drug combination had the potential to opsonize tumor cells to the novel immunotherapy [22,23]. With both [neratinib + HDAC inhibitors] and [pemetrexed + sildenafil], as well as with other regimens which strongly induced autophagosome formation, we observed that drug-treated cells rapidly reduced their expression of PD-L1, ODC and IDO-1, and increased their expression of Class I MHCA [24,25]. Our initial rationalization of these findings was that altered cell signaling processes, including transcription factors, likely played a key role in altered protein expression. However, in the case of cells exposed to pemetrexed and sildenafil, we observed that drug-induced activation of ataxia telangiectasia (ATM) regulated autophagosome formation via the AMP-dependent protein kinase (AMPK) and simultaneously also caused the inactivation of the ATPase activity of the chaperone HSP90. Inactivation of the HSP90 ATPase was associated with increased acetylation of the chaperone and decreased expression of the HDAC which deacetylates HSP90, HDAC6 [26,27]. HDAC6 and HSP90 were degraded via autophagosome formation. Reduced HSP90 function was shown to enhance the levels of denatured proteins, which in the endoplasmic reticulum, is sensed by the chaperone GRP78 and PKR-like endoplasmic reticulum kinase (PERK) [28,29]. GRP78 dissociates from PERK to chaperone and renature the denatured proteins, causing PERK to become activated and to phosphorylate eIF2α; phosphorylation of eIF2 α results in the translation of most mRNA molecules to decline, though for some genes such as the autophagosome-regulatory ATG5 and Beclin1, the reverse is true [30,31].

HDAC6 was being degraded by autophagy and in our extensive screening studies examining signal transduction pathways, no obvious association could be made between the rapid drug-effects on the pathways or transcription factors and the rapid alterations in the expression of PD-L1, MHCA, ODC or IDO1 [16–29]. Hence, we determined whether the pemetrexed and sildenafil combination or the neratinib and HDAC inhibitor combination altered the protein expression of individual HDAC proteins (HDACs1–11). Over a series of research manuscripts, we discovered that multiple HDAC proteins were susceptible to degradation via drug-induced autophagy, e.g., [17,23–25]. HDACs1–3 and HDAC6 were consistently demonstrated, between cell types and different drug combinations, to have their expression reduced via autophagosome formation [16–25].

It is well-known that HDAC inhibitors can influence the biology of the immune cells outside and within a tumor [14,32–34]. In general, HDAC inhibitors are viewed as acting in a positive fashion, increasing the activity of immune cells, including T cells and macrophages [35,36]. Hence it would be expected that HDAC inhibitors alone, or HDAC inhibitors combined with neratinib would very probably also enhance the anti-tumor efficacy of the immune system. The obvious next steps were to mechanistically define whether the altered expression of HDAC proteins was causal in the altered expression of the immunogenic regulatory proteins. In a cell-type-dependent fashion, combined knock down of HDAC1 and HDAC2, HDAC1 and HDAC3, or HDAC2 and HDAC3 prevented the various drug exposures from decreasing PD-L1 levels or enhancing Class I MHCA expression. Thus, using multiple drug combinations, all of which strongly promote autophagosome formation, we determined that protein metabolism/degradation of HDACs results in enhanced or suppressed expression of key proteins involved in regulating tumor cell immunogenicity.

## *IN VIVO* ASSESSMENTS OF TUMOR CELL IMMUNOGENICITY AFTER EXPOSURE TO DRUG COMBINATIONS WHICH PROMOTE AUTOPHAGOSOME FORMATION

Our *in vitro* data experiences with a diverse cohort of different drug combinations that all rapidly induce autophagosome formation, endoplasmic reticulum stress signalling and HDAC metabolism would all a priori predict for enhanced tumor cell immunogenicity in pre-clinical mouse models. Below we discuss some of our findings.

## LEWIS LUNG CARCINOMA TUMOURS

In Lewis Lung Carcinoma tumours [22,23] pemetrexed and sildenafil interacted to further supress tumor growth, which was then enhanced by administration of either anti-PD1 or anti-CTLA4 checkpoint inhibitory antibodies. These findings validate the *in vitro* experience of observing autophagy-dependent reduced PD-L1 and enhanced MHCA expression. [Pemetrexed + sildenafil] exposure increased the levels of infiltrating macrophages within the tumor, with the majority belonging to the M1 subtype. This was additionally increased when tumors had been treated with an anti-PD-1 antibody. Individually, either an anti-PD-1 antibody or to [pemetrexed + sildenafil] modestly increased natural killer cell levels in the tumors and combined exposure to the agents profoundly enhanced NK levels. Similar data to that for NK cells was also observed for neutrophils. [Pemetrexed + sildenafil] or to an anti-PD-1 antibody increased T cell levels, including activated T cells, within the LLC tumors. The combination of both agents further elevated T cell infiltration, causing T cells to localize into the parenchyma of the tumor. Of particular note, was that approximately fourteen days after drug exposure, the levels of both PD-L1 and HDAC6 were still significantly lower than those observed in the vehicle control treated tumors and also that the expression of MHCA continued to be elevated. In other words, the actions of [pemetrexed + sildenafil] had re-programed the surviving LLC cells to have a maintained higher immunogenicity for checkpoint inhibitory antibodies.

## B16 MELANOMA TUMORS

In B16 melanoma tumors [25]; the HDAC inhibitors AR42 or sodium valproate enhanced the efficacy of both anti-PD1 or anti-CTLA4 antibodies. Prior [pazopanib + HDAC inhibitor] exposure caused higher levels of F4/80+ macrophage infiltration who expressed inducible nitric oxide synthase (iNOS), *i.e*., the M1 macrophage phenotype. Exposure of B16 tumors to HDAC inhibitors or to an anti-PD-1 antibody elevated the tumor numbers of NK cells, neutrophils and activated T cells. Combined treatment of tumors to the HDAC inhibitor and an anti-PD-1 antibody caused a dispersion of the macrophages, NK cells, neutrophils and activated T cells within the tumor. Multiplex antibody array assays were performed on the plasma of animals carrying B16 tumors, that had been treated with an [HDAC inhibitor + anti-PD-1 antibody]. The plasma exhibited elevated levels of IL-6, IL-12, CCL2, CCL3, CCL5, CXCL9 and CXCL2 and reduced IL1a levels. Elevated levels of IL-6, IL-12, CCL2, CCL3, CCL5, CXCL9 would *a priori* predict for a greater infiltration of the tumor by M1 type macrophages, which independently confirms our prior IHC staining findings.

## 4T1 MAMMARY CARCINOMA TUMORS

In 4T1 mammary carcinoma tumors [17–19]; neratinib and the HDAC inhibitor sodium valproate interacted to further supress mammary tumor growth, which was further enhanced by administration of either anti-PD1 or anti-CTLA4 antibodies. [Neratinib + sodium valproate] treated tumors, two weeks after the last drug exposure, still exhibited a significantly reduced expression of K-RAS, N-RAS and ERBB1. At higher 60× magnification we discovered that vehicle control tumors stained for RAS proteins that were localized at the periphery of the cell, but in tumors previously exposed to [neratinib + valproate] the much feebler staining for RAS presented as being evenly distributed all over the cell. [Neratinib + valproate] treatment significantly lowered the expression of IDO-1 and ODC which was not modified in tumors that were also exposed to the anti-PD1 antibody as a single agent. [Neratinib + valproate] treatment elevated the expression of Class I MHCA and lowered the expression of PD-L1. Reduced expression of PD-L1 associates with the increased anti-tumor efficacy of the anti-PD1 antibody. Treatment with the anti-PD1 antibody strongly lowered PD-L1 expression in the 4T1 tumors, and the drug combination with the antibody additionally enhanced MHCA levels.

[Neratinib + valproate] exposure enhanced M1 polarized macrophage and M2 polarized macrophage levels in the tumor. For M1 macrophages, 80% of the F4/80 staining cells co-localized with iNOS but only 20% of the F4/80 staining cells co-localized with the M2 macrophage biomarker arginase. [Neratinib + valproate] treatment caused enhanced infiltration of immune cells that stained strongly for CD69 and CD335, *i.e*., activated natural killer cells. CD69^+^ CD335^+^ levels were further enhanced when an anti-PD1 antibody was added to [neratinib + valproate]. [Neratinib + valproate] exposure increased CD8^+^ T cell and CD69^+^ CTLA4+ activated T cell levels. Hence, exposure to [neratinib + valproate] causes anti-tumor immune responses via multiple overlapping mechanisms; infiltrating NK cells, M1 macrophages and reduced IDO-1 and ODC levels. Exposure of tumors with [neratinib + valproate] caused a significant decline in the levels of HDACs 1–3, 6 and 10 in the tumor. Hence, not only does [neratinib + valproate] reduce HDAC expression *in vitro* in the short term (hours), but the combination re-programs the surviving tumor cells *in vivo* to express less of these HDACs in tumors over a longer time period (weeks).

## PAN02 PANCREATIC TUMORS

In PAN02 pancreatic tumors [24]; prior exposure of the tumours to the multi-kinase inhibitor sorafenib and the HDAC inhibitor vorinostat enhanced the efficacy of an anti-PD1 antibody. Twenty days after the end of any sorafenib/vorinostat/antibody exposure the levels of K-RAS in the tumor cells remained significantly reduced, as was ERBB1expression. The level of IDO-1 was significantly lower in tumors exposed previously to [sorafenib + vorinostat] but on the other hand, anti-PD-1 antibody exposure enhanced the levels of IDO-1. The total expression of ERK2, which remains invariant during *in vitro* drug exposures, remained constant under all *in vivo* treatment conditions. Treatment of PAN02 tumors with [sorafenib + vorinostat] did not significantly change the numbers of CD4^+^ T cells, however the numbers of CD8^+^ T cells were significantly elevated, with the drug combination plus anti-PD-1 antibody significantly enhancing CD8^+^ cell numbers beyond either individual treatment. [Sorafenib + vorinostat], as had previously been shown *in vitro*, had lowered the levels of PD-L1 and elevated MHCA levels. The alterations in biomarker levels and immune cell invasion were associated with an increased anti-PD-1 antibody efficacy when added to the [sorafenib + vorinostat] drug combination.

Macrophage invasion into a drug-treated tumor plays an essential role in the outcome of anti-cancer therapies. M2 polarized macrophages express high levels of IL-10 and TGF beta, and relatively lower levels of IL-12. M1 polarized macrophages express reduced levels of IL-10 and elevated levels of IL-12. [Sorafenib + vorinostat] exposure lowered the expression of IL-10 and TGFβ in both the tumor and the immune cell infiltrate. The levels of IL-10 and TGF beta were further enhanced by addition of an anti-PD-1 antibody. However, [sorafenib + vorinostat] exposure significantly increased IL-12 levels in the tumor and in the immune cell infiltrate. The chaperone HSP90 is regulated by acetylation, via the actions of a HAT and HDAC6. [Sorafenib + vorinostat] exposure lowered the expression of HDAC6 by >60% and increased the phosphorylation of eIF2α S51 by >40%. Although eIF2α phosphorylation was enhanced, the level of GRP78 was not decreased in tumors or immune cells exposed to [sorafenib + vorinostat] or to the anti-PD-1 antibody. HSP90 expression was significantly lower in tumors treated with [sorafenib + vorinostat] with the anti-PD-1 antibody.

[Sorafenib + vorinostat] exposure enhanced F4/80+ staining of cells within the tumor as well as of iNOS, an M1 polarization marker, and arginase, an M2 polarization marker. The F4/80 and iNOS stains co-localized, arguing for M1 macrophage invasion. However, the F4/80 and arginase staining did not co-localize. Elevated levels of arginase within pancreatic cancer cells is toxic. Treatment of tumors with [sorafenib + vorinostat] and an anti-PD-1 antibody promoted a greater than additive increase in the levels of F4/80 + iNOS + M1 macrophages within and at the leading edge of the tumors. The numbers of iNOS staining cells were enhanced whereas the numbers of arginase staining cells was reduced below baseline levels.

[Sorafenib + vorinostat] exposure enhanced CD69^+^ CD335^+^ co-staining of cells, *i.e*., active natural killer cells, in the center and at the leading invasive edge of tumors. Furthermore, treatment with the drug combination and an anti-PD-1 antibody promoted an additional increase in NK cell levels within the tumor. [Sorafenib + vorinostat] treatment also increased the number of CD11b staining cells but lowered staining for GR-1. These findings argue that the numbers of myeloid derived suppressor cells (MDSCs) were decreased.

In multiplex antibody arrays assays the majority of cytokines in tumor tissue and mouse plasma were alike, with the exception of IL-16, CCL12 and CCL3 whose expression was increased in tumor tissues. Compared to control-treated tumors, those previously treated with [sorafenib + vorinostat] + anti-PD-1 displayed lower concentrations of CXCL-13, CCL27, CXCL-5, CCL11, KC, G-CSF, GM-CSF, interferon γ, IL-1α, IL-1β, IL-2, IL-3, IL-4, IL-5, IL-6, IL-10, IL-13, CXCL10, CXCL-1, CCL7, CCL22, CCL4, CCL5, CXCL-16, CCL17, CCL25 and TNFα. The expression of IL-12 p40 and IL-12 p70 were increased in the drug exposed tumors. In the plasma, the amount of CXCL-13, CXCL-5, IL-16, CCL7, CXCL-16 and CXCL-12 were increased. No significant reductions in the levels of any plasma cytokine were observed.

## CONCLUSIONS

In multiple manuscripts we have demonstrated that drugs and especially drug combinations which strongly enhance autophagosome formation and autophagic flux reduce the expression of multiple HDAC proteins, and that the reduced levels of the HDACs coordinately result in decreased expression of PD-L1, ODC and IDO1, and increased levels of Class I MHCA. These changes in protein expression resulted in tumor cells that were more readily killed *in vivo* by checkpoint inhibitory immunotherapy antibodies. Of the tumor types tested, two have approved immunotherapy modalities, lung and melanoma, and two do not, breast and pancreatic. Hence, the approach of altering HDAC expression may be useful as a neo-adjuvant to improve the immunotherapy responses of tumor types considered to be “cold”.

The AMPK and mTOR are well-recognized regulators of cellular metabolism, and one down-stream component of their biology is to control the process of autophagy. Throughout evolution autophagy is a survival process, degrading cellular components to sustain viability. In tumor cells, our research has shown that autophagy can degrade a diverse variety of cellular proteins, including HDACs. Thus, the regulation of cell metabolism, *i.e*., AMPK and mTOR, intersects with HDAC protein expression which in turn alters transcription, resulting in enhanced levels of MHCA and decreased levels of PD-L1 which enhances the efficacy of checkpoint inhibitory antibodies. These findings raise the possibility of developing novel agents to enhance autophagic flux, thereby increasing tumor cell immunogenicity.

## ABBREVIATIONS

ERK: extracellular regulated kinase
PI3K: phosphatidyl inositol 3 kinase
ER: endoplasmic reticulum
HDAC: histone deacetylase
AMPK: AMP-dependent protein kinase
mTOR: mammalian target of rapamycin
JAK: Janus Kinase
STAT: Signal Transducers and Activators of Transcription
MAPK: mitogen activated protein kinase
PTEN: phosphatase and tensin homologue on chromosome ten
ROS: reactive oxygen species
PD-L1: programed death ligand 1
ODC: ornithine decarboxylase
IDO1: Indoleamine 2,3-dioxygenase
MHCA: Human Major Histo-compatibility Complex Class I A
CTLA4: cytotoxic T-lymphocyte-associated protein 4
GRP78: glucose regulated protein 78
mTOR: mammalian target of rapamycin
IRE1: inositol-requiring enzyme 1
